# Kaempferol-loaded bioactive glass-based scaffold for bone tissue engineering: in vitro and in vivo evaluation

**DOI:** 10.1038/s41598-023-39505-8

**Published:** 2023-07-31

**Authors:** Faezeh Esmaeili Ranjbar, Saeed Farzad-Mohajeri, Saeed Samani, Jamileh Saremi, Rahele Khademi, Mohammad Mehdi Dehghan, Mahmoud Azami

**Affiliations:** 1 Molecular Medicine Research Center, Research Institute of Basic Medical Sciences, Rafsanjan University of Medical Sciences, Rafsanjan, Iran; 2 Department of Surgery and Radiology, Faculty of Veterinary Medicine, University of Tehran, Dr. Qarib Street, Azadi Street, Tehran, 1419963111 Iran; 3 Department of Tissue Engineering, School of Advanced Technologies in Medicine, Tehran University of Medical Sciences, No. 88, Italia St., Keshavarz Blv, Tehran, Iran; 4 Research Center for Noncommunicable Diseases, Jahrom University of Medical Sciences, Jahrom, Iran

**Keywords:** Bioinspired materials, Biomaterials - cells

## Abstract

Due to the increasing prevalence of bone disorders among people especially in average age, the future of treatments for osseous abnormalities has been illuminated by scaffold-based bone tissue engineering. In this study, in vitro and in vivo properties of 58S bioactive glass-based scaffolds for bone tissue engineering (bare (B.SC), Zein-coated (C.SC), and Zein-coated containing Kaempferol (KC.SC)) were evaluated. This is a follow-up study on our previously published paper, where we synthesized 58S bioactive glass-based scaffolds coated with Kaempferol-loaded Zein biopolymer, and characterized from mostly engineering points of view to find the optimum composition. For this aim, in vitro assessments were done to evaluate the osteogenic capacity and biological features of the scaffolds. In the in vivo section, all types of scaffolds with/without bone marrow-derived stem cells (BMSC) were implanted into rat calvaria bone defects, and potential of bone healing was assessed using imaging, staining, and histomorphometric analyses. It was shown that, Zein-coating covered surface cracks leading to better mechanical properties without negative effect on bioactivity and cell attachment. Also, BMSC differentiation proved that the presence of Kaempferol caused higher calcium deposition, increased alkaline phosphatase activity, bone-specific gene upregulation in vitro. Further, in vivo study confirmed positive effect of BMSC-loaded KC.SC on significant new bone formation resulting in complete bone regeneration. Combining physical properties of coated scaffolds with the osteogenic effect of Kaempferol and BMSCs could represent a new strategy for bone regeneration and provide a more effective approach to repairing critical-sized bone defects.

## Introduction

Bone losses or failures are important problems in medicine which could influence on life quality of human beings^1^. Despite self-regenerative ability of the bone to repair small damages without production of fibrotic tissue, the defects larger than critical size cannot be repaired independently^2^. Autografts, allografts, and xenografts are traditional methods for bone replacement among which autograft implants are known as the gold standard for bone regeneration. Considering important limits for traditional methods the tissue engineering (TE) approach has emerged as an alternative applicable suggestion for tissue regeneration and replacement^3–5^.

Tissue engineering is an interdisciplinary field for maintaining, improving, and regenerating lost tissues and organs. Three-dimensional porous scaffolds as the main part of a tissue engineering system with or without cells and stimulating factors are important in bone tissue engineering (BTE). These scaffolds should be biocompatible, osteoinductive, osteoconductive, and biodegradable and encourage osteoprogenitor cells to migrate, attach, grow and differentiate into osteoblast cells^4–7^. Replacing bone defects with three-dimensional and porous structures can induce bone regeneration^8^.

From developing bioactive glass (BG) by Larry Hench in 1969^9^, many studies have used BGs as the raw material to prepare scaffold for bone regeneration^10–13^. Bioactive glass could bonds to soft and hard tissues without fibrous tissue formation, and produces osteoconductive, osteoinductive, bioactive degraded products^14–17^. The 58S bioactive glass, a three-component system containing 58% SiO_2_, 38% CaO, and 4% P_2_O_5_, has recently received more attention because of its outstanding biological characteristics. It can be degraded and release Si, Ca, and P ions that enhance bonding with bone tissue, specific gene expressions, and osteoblast growth without inflammatory or foreign-body reactions^18–20^. In contrast to quick and long-lasting alkalinization made by 45S5 bioglass, the 58S bioglass particles do not change the pH of its surrounding medium, and has not negative effect on cell survival^18,21^. Moreover, 58S bioglass can be prepared by melt-derived and sol–gel method. In contrast to melt-derived as a traditional synthesis method, the sol–gel method is an excellent alternative procedure performing at low temperature and providing higher purity, homogeneity, biodegradability and bioactivity^22,23^. Overall, 58S bioactive glass produced by sol–gel method was selected as the raw material for our basic scaffold^16,24^.

Bioactive glasses, similar to other bioceramics, are brittle and have weak mechanical properties that catch more concerns for porous tissue-engineered scaffolds. Many studies have aimed to apply various approaches to strengthen the structure^25^ among which surface coating with a suitable polymer could improve mechanical strength by filling and covering surface micro-cracks^26^. Besides, the polymer coating could act as a carrier for sustained drug release^27–29^.

Natural polymers such as chitosan, starch, alginate, collagen, gelatin, and silk have been recently used for coating on biomedical devices because of their biocompatibility and biodegradability^18,30,31^. Zein, a natural bioactive and antioxidant polymer came from corn^32,33^, is a biocompatible and biodegradable polymer which has been used in many studies as the scaffold for bone regeneration^34–36^. Also, reported findings have showed that Zein is a suitable biopolymer in pharmaceutical applications, food packaging, and sustained drug release^37^. Zein is a plant-based protein that has some advantages compared to synthetic polymers and commonly used animal proteins. It is biocompatible, relatively inexpensive, safe, and able to be produced from renewable sources^38^.

Kaempferol is an available yellow flavonoid powder^39,40^ which could act as an osteogenic, anti-osteoclastogenic, and antiosteoporotic agent performing through different signaling pathways^40,41^. Due to importance of local drug delivery for BTE^42,43^, we developed a 58S-based scaffold coated with Kaempferol-loaded Zein having improved mechanical strength and sustained Kaempferol release^44^.

Mesenchymal stem cells (MSCs) are multipotent stem cells that can differentiate into various cell types, and support regeneration of injured tissues^45–47^. Considering immunomodulatory potency^48^ and positive effect on bone healing, MSCs are excellent candidate for bone tissue engineering^49^.

In our previous study^44^, we prepared 58S-based scaffolds by foam replication method, coated with Kaempferol-containing Zein solution, and characterized for engineering properties. Based on our findings, scaffolds were biocompatible, bioactive and biodegradable (about 20% weight loss during 60 days). Moreover, Zein coating did not prevent cell adhesion, and improve mechanical strength. After finding a good-designed scaffold as a possible bone substitute for tissue engineering, current study focused on evaluation of its biological performance from in vitro and especially in vivo points of view. For this aim, we prepared coated and uncoated 58S-based scaffolds with or without bone marrow-derived stem cells (BMSCs) and assessed the cell attachment, differentiation and bone regeneration using different in vitro and in vivo methods.

## Results

In this study, we made bare (uncoated, B.SC) and Zein-coated 58S bioglass-based scaffolds without/with Kaempferol loading (C.SC or KC.SC) to offer mechanical augmentation by Zein and osteogenesis by Kaempferol to the scaffolds, and assessed them from biological and osteogenesis point of view in vitro and in vivo. Moreover, the scaffolds were loaded with bone marrow stem cells (BMSCs) to amplify osteogenesis capacity.

### Scaffold preparation and engineering characterization

**Figure 1:**
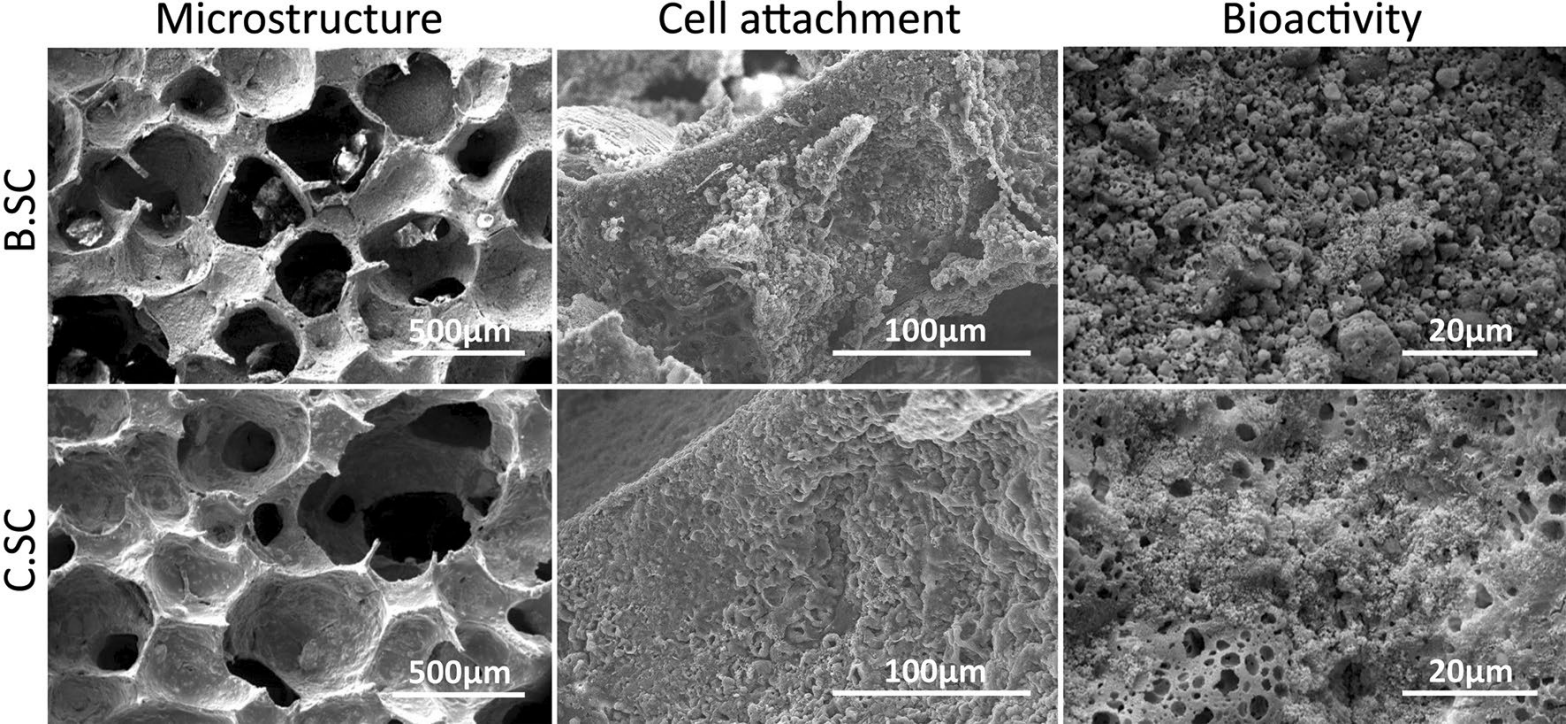
SEM images showing the morphology, bioactivity, and cell adherence (BMSC after 5 days incubation) of B.SC and C.SC scaffolds.

Figure 1 shows the surface structure, cell attachment, and bioactivity of bare and coated scaffolds. According to scanning electron microscope (SEM) images of scaffolds, Zein coating (7% (w/v)) covered surface and filled micro-cracks without filling surface micropores. Cell attachment experiment proved that MG-63 cells could attach and spread on bare and Zein-coated scaffolds as well. Bioactivity assessment of scaffolds was done through immersion in SBF for 14 days. The SEM images showed hydroxy carbonate apatite (HCA) particles covered bare and Zein-coated scaffold and coating had not negative effect on bioactivity. Also, Zein coating at concentration of 7% (w/v) could improve compressive strength (3.06 MPa) compared with bare scaffold (0.88 MPa)^44^.

### Characterization of bone marrow-derived mesenchymal stem cells (BMSCs)

**Figure 2:**
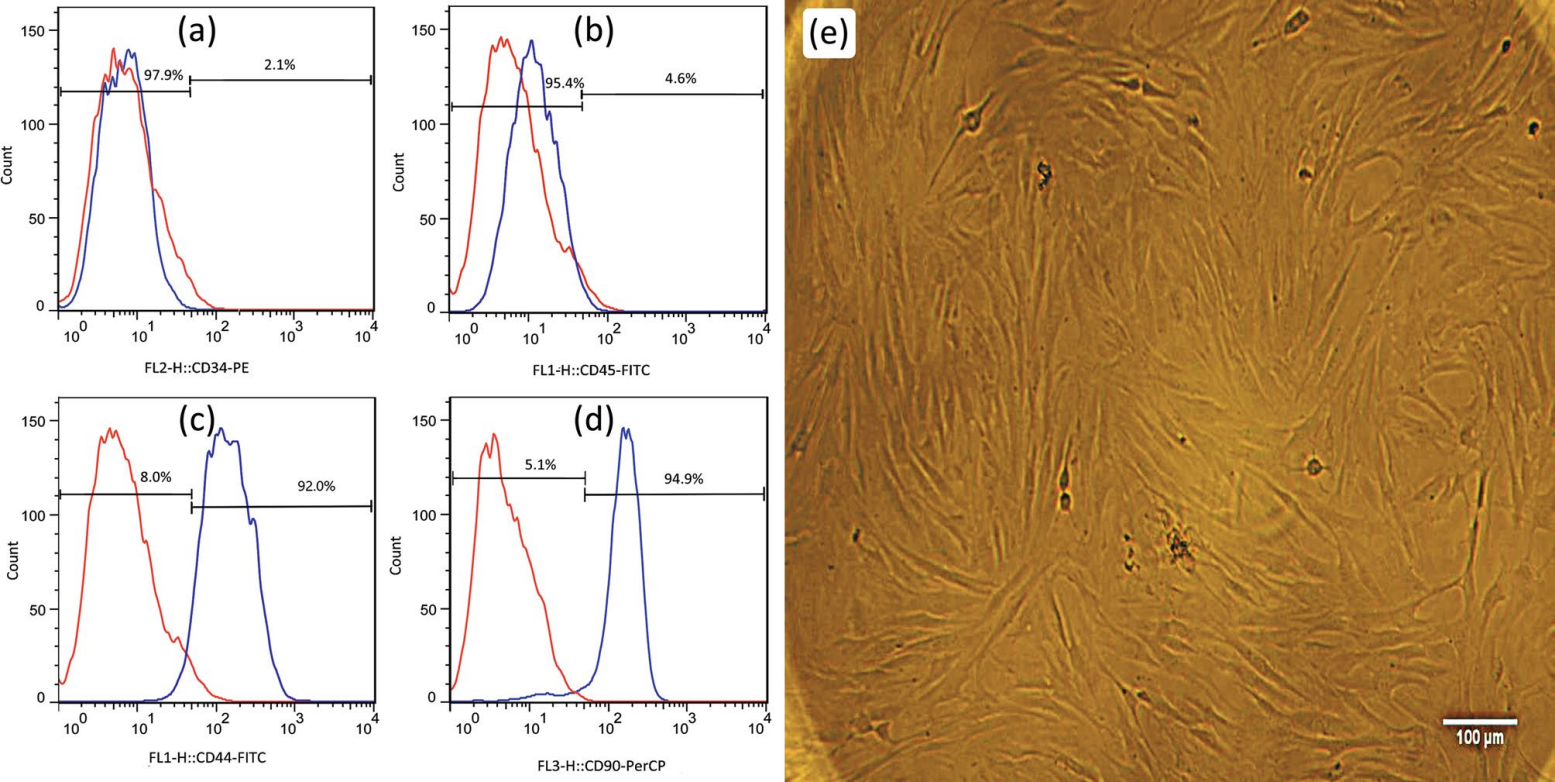
Rat BMSCs characterization, these cells were (**a** and **b**) negative for CD34 (97.9%) and CD45 (95.4%), and (**c** and **d**) positive for CD90 (94.9%), CD44 (92%) and markers. Red and blue peaks refer to control and antibody-treated sample respectively (**e**) the inverted light microscope images indicates BMSCs cell.

Isolated BMSCs (passage 3) were evaluated by flow cytometry based on specific surface markers whose result reported as Fig. 2a-d. Analysis showed that BMSCs were positive for CD90 and CD44 markers and negative for CD34 (endothelial marker) and CD45 (Hematopoietic marker) markers. Figure 2e shows the optical microscopy image of isolated BMSCs.

### In vitro assessments of biological activity and gene expression

#### Calcium deposition assessment

**Figure 3:**
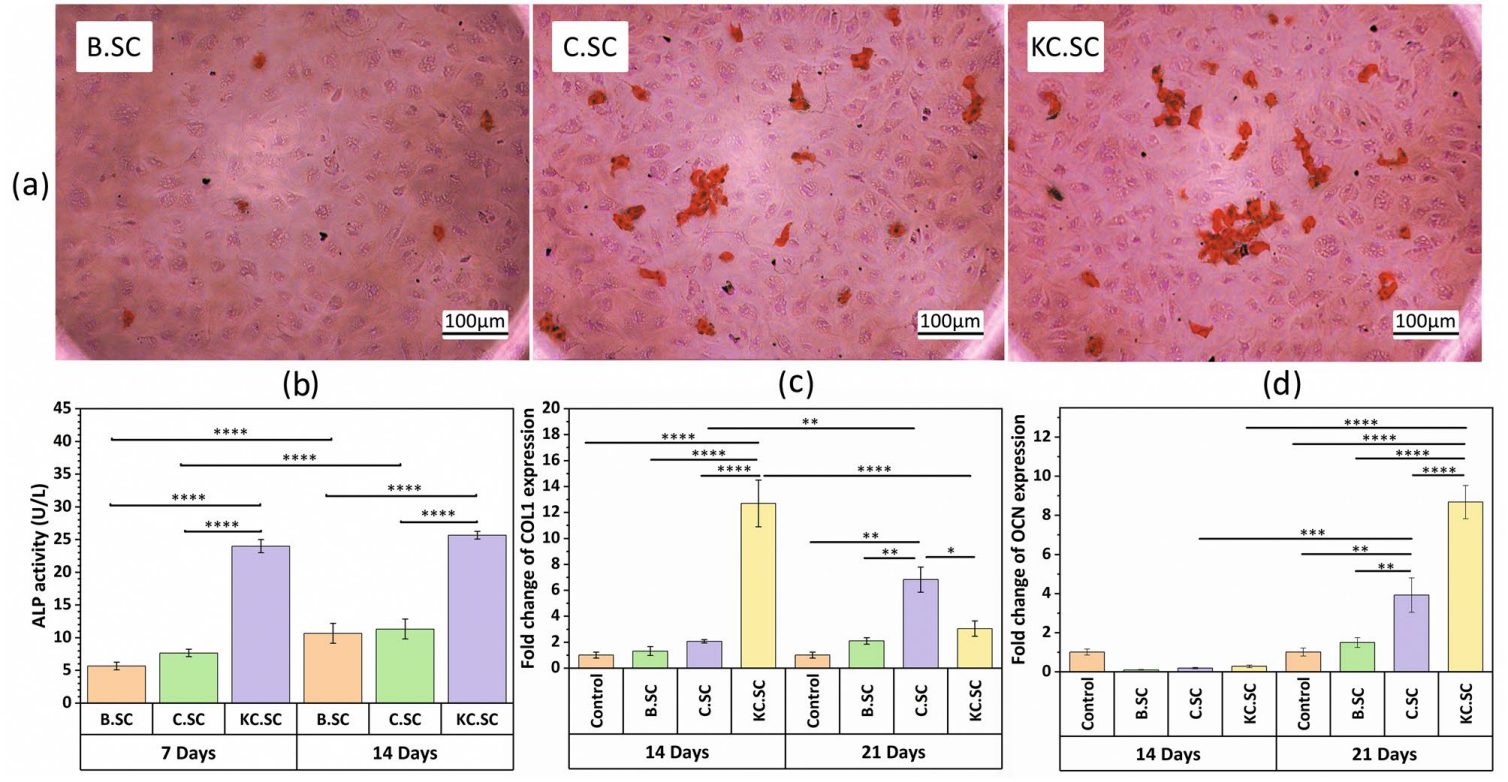
(**a**) Alizarin red S staining of mineral deposition on ECM in B.SC, C.SC, and KC.SC groups after 21 days incubation (scale bar = 100 μm). (**b**) ALP activities for B.SC, C.SC, and KC.SC after 7-days and 14-days treatment with conditioned media. Diagrams show the relative expressions of (**c**) COL1 (Early marker of osteogenic differentiation) and (**d**) OCN (a late marker of osteogenic differentiation) 14 and 21 days post-incubation in the scaffolds extraction respectively. An asterisk indicates the statistically significant difference between genes and control as measured by Student’s t-test (*p < 0.05, **p < 0.01, ***p < 0.001, ****p < 0.0001).

Osteoblast-like differentiated cells started to deposit calcium in their extra cellular matrix (ECM) recognized by alizarin red S staining. After 21-days treatment with conditioned media, deposition of calcium nodules in C.SC and KC.SC was higher than B.SC and the best calcium deposition was occurred because of Kaempferol in KC.SC scaffold (Fig. 3a).

#### Alkaline phosphatase (ALP) activity

To estimate the early differentiation of BMSCs to osteoblast-like cells, BMSCs cultured in conditioned media and their ALP activity were measured after 7 and 14 days. The result (Fig. 3b) revealed that Kaempferol (KC.SC sample) could increase ALP activity considerably after each time point compared with B.SC and C.SC samples. Although the ALP activities of B.SC and C.SC samples after 14-days culturing were higher than ALP activities after 7-days culture, time passage did not have a significant effect on the ALP activity of the KC.SC sample.

#### Bone-specific gene expression

The osteogenic effect of B.SC, C.SC, and KC.SC on BMSCs was analyzed by evaluating expression of COL1 and OCN genes after 14 and 21 days using real time-PCR. Results (Fig. 3c) proved that the COL1 gene was upregulated after 14 days in KC.SC group compared with other groups. However, the COL1 expression of the C.SC sample was enhanced after 21 days compared with B.SC and control samples and the 14-days same sample. Further, the OCN gene as a late marker was upregulated significantly in C.SC and KC.SC groups after 21 days compared with other groups (Fig. 3d).

### Scaffold transplantation and in vivo study

To assess osteogenesis and bone regeneration, critical-sized defects were created in the rat calvarias and various types of scaffolds with or without BMSCs were transplanted. Afterwards, regeneration potential was investigated using radiography, µCT, and cell staining after 4 and 12 weeks.

#### ***Radiological and three-dimensional micro-computed tomography (3D μCT)***

**Figure 4:**
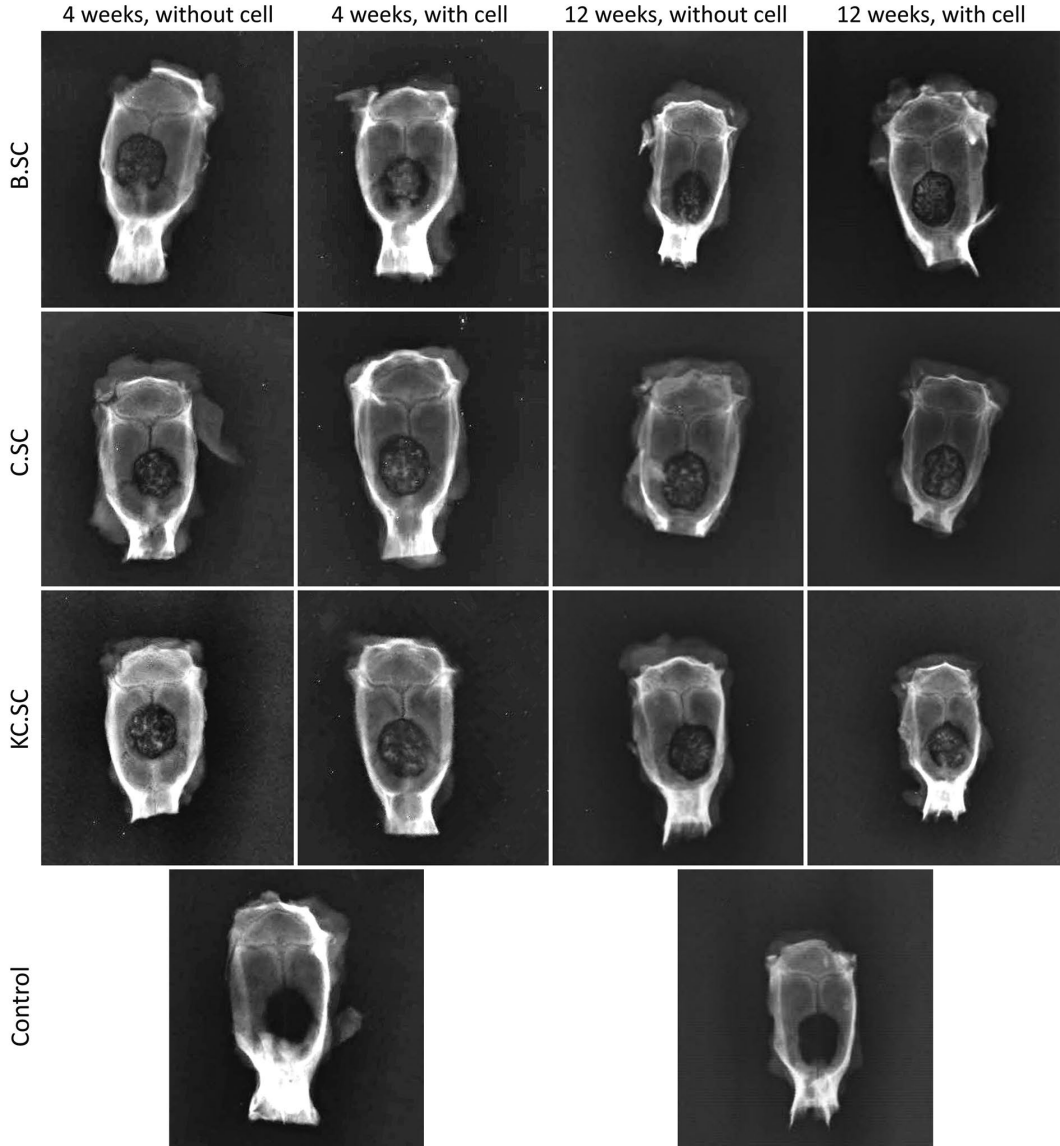
Reprehensive radiographs after 4 and 12 weeks post-implantation. B.SC: bare scaffold, C.SC: Zein-coated scaffold, KC.SC: Kaempferol-loaded C.SC, B.SC + cell: bare scaffold + BMSC, C.SC + cell: Zein-coated scaffold + BMSC, KC.SC + cell: Kaempferol-loaded KC.SC + BMSC.

**Figure 5:**
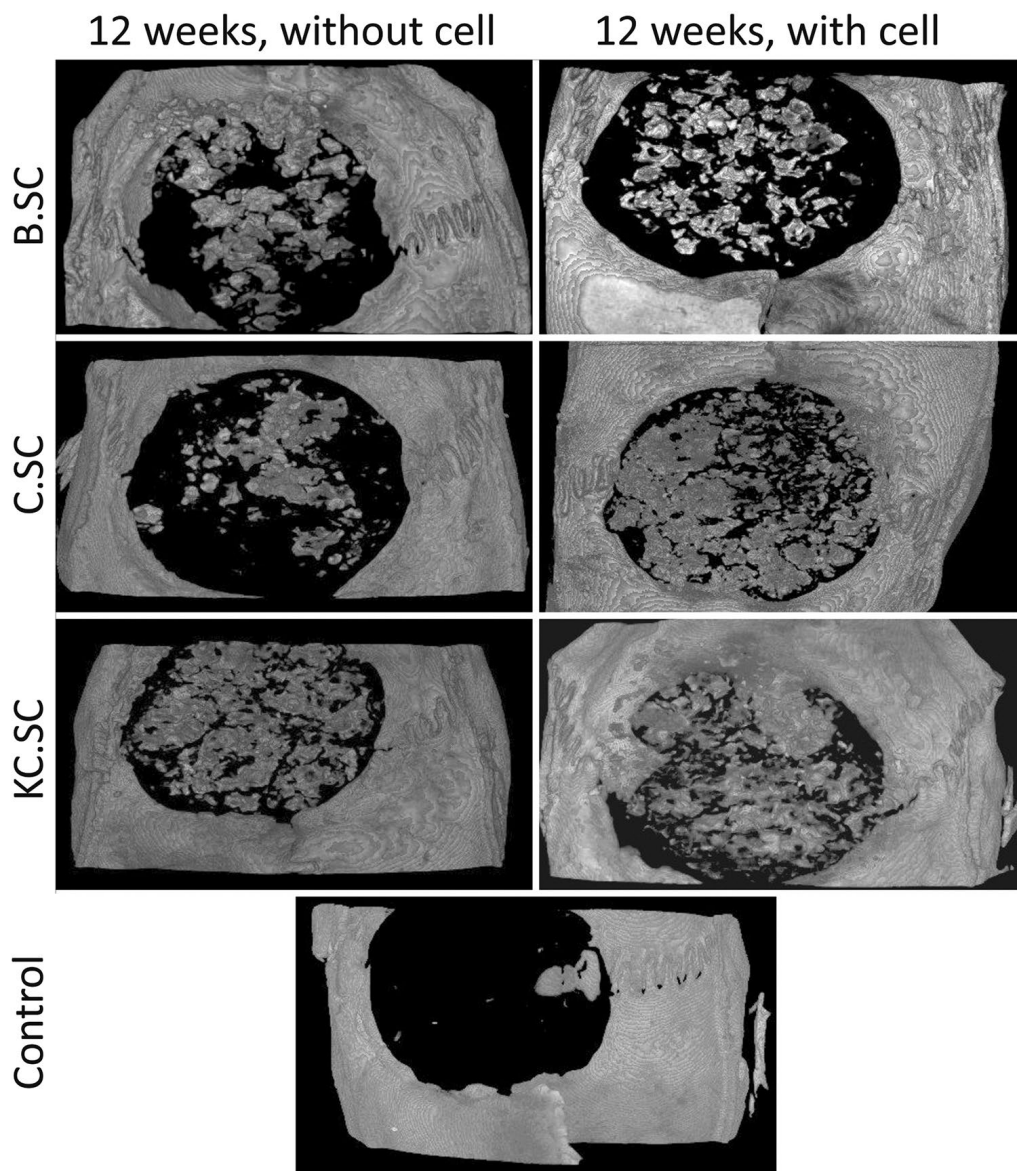
µCT imaging shows bone regeneration in calvaria defects after 12 weeks.

Figure 4 shows radiographic images of the rat calvarias 4 and 12 weeks post-implantation. Considering radio-opacity caused by mineral deposition, bone regeneration was not observed in the control group. All 58S-based scaffolds especially cell-loaded ones could improve bone formation after 4 and 12 weeks and BMSC presence had great influence on scaffold-bone integration. The µCT analysis (Fig. 5) confirmed the capacity of various scaffolds for bone formation especially at the edge of calvaria by cell-loaded C.SC and KC.SC scaffolds.

#### Histomorphometric analysis

**Figure 6:**
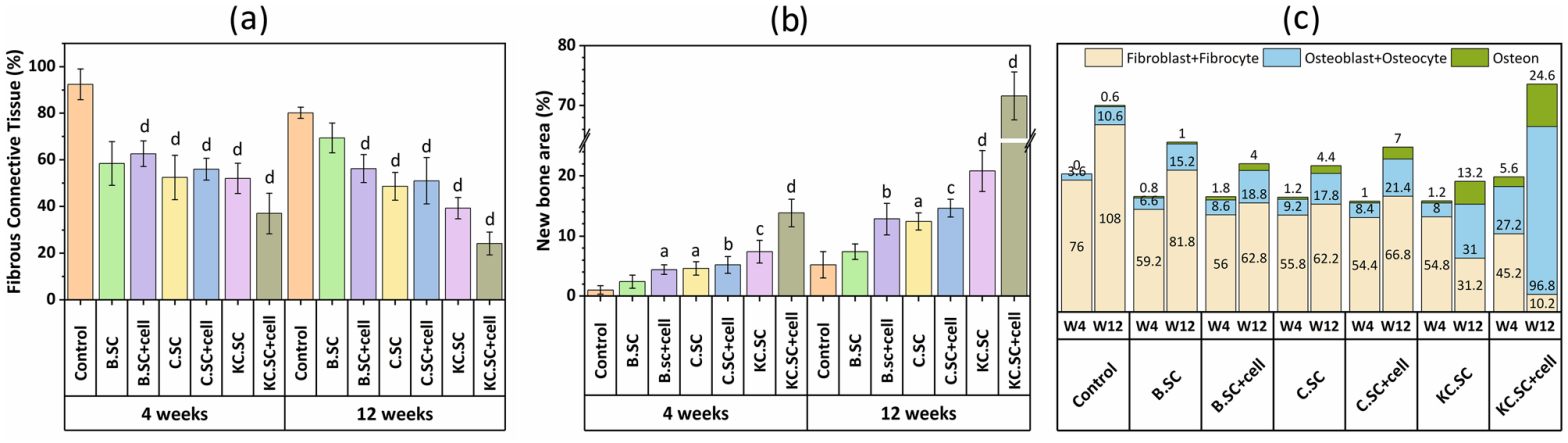
Histomorphometric analysis after 4 and 12 weeks of healing.

Figure 6 shows quantitative histomorphometric analysis including percentages of fibrous connective tissue (FCT%, Fig. 6a), new bone area (NB%, Fig. 6b), and number of cells and osteons (Fig. 6c). After evaluation periods (4 and 12 weeks), although all 58S-based scaffolds could improve bone formation compared with the control group, it was obvious that BMSC-loaded KC.SC had the best effect whose NB% increased by ~ 200% after 12 weeks. However, almost all scaffolds could prevent formation of fibrous connective tissue and BMSC-loaded KC.SC had the lowest FCT% among all investigated groups. As seen in Fig. 6c, the number of osteoblasts, osteocytes, and osteons were the highest in KC.SC + cell groups compared with other groups after 12 weeks. On the contrary, the highest number of fibroblasts belonged to the control group.

#### Immunohistochemical analysis

**Figure 7:**
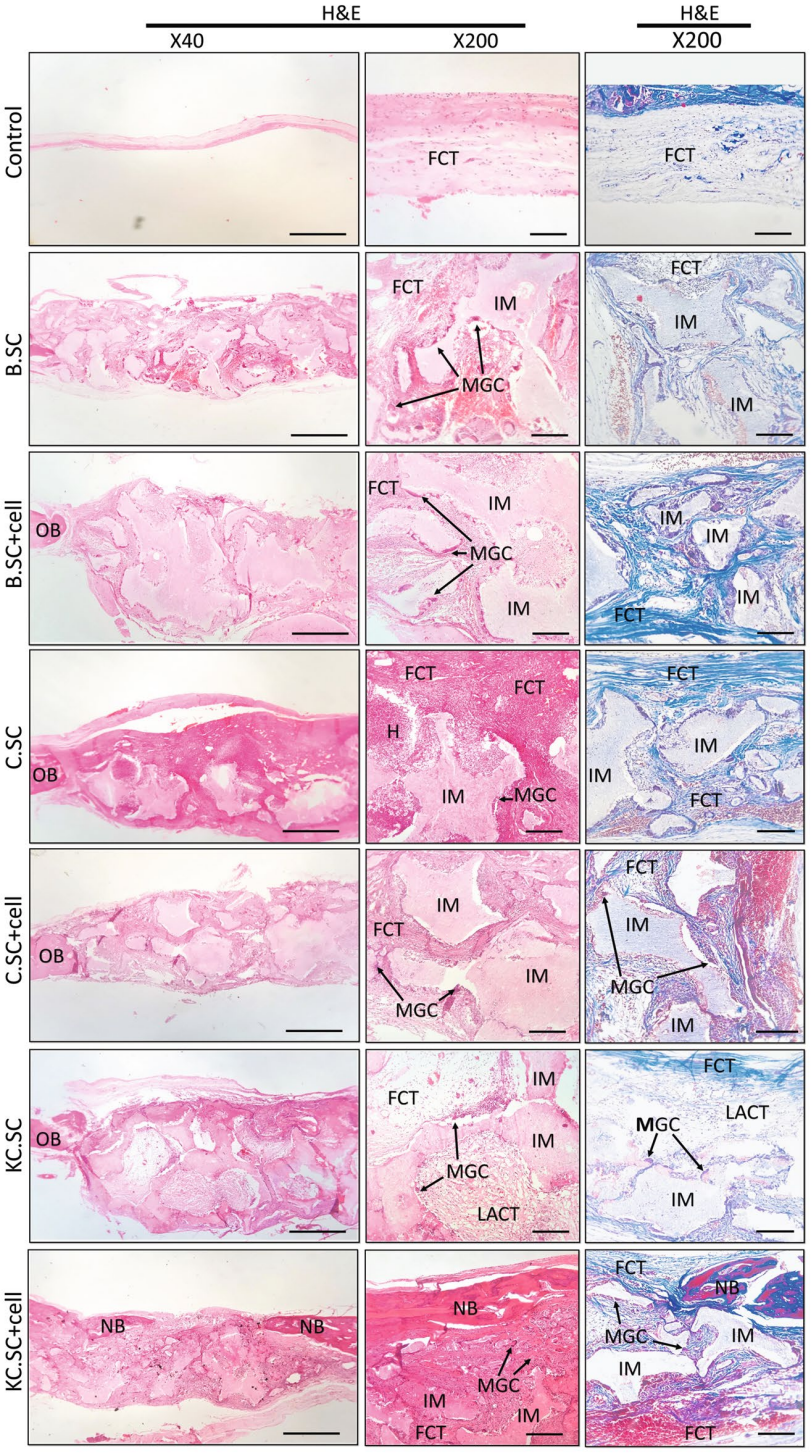
Histopathology images (containing H&E and MT staining), 4 weeks after implantation.

**Figure 8:**
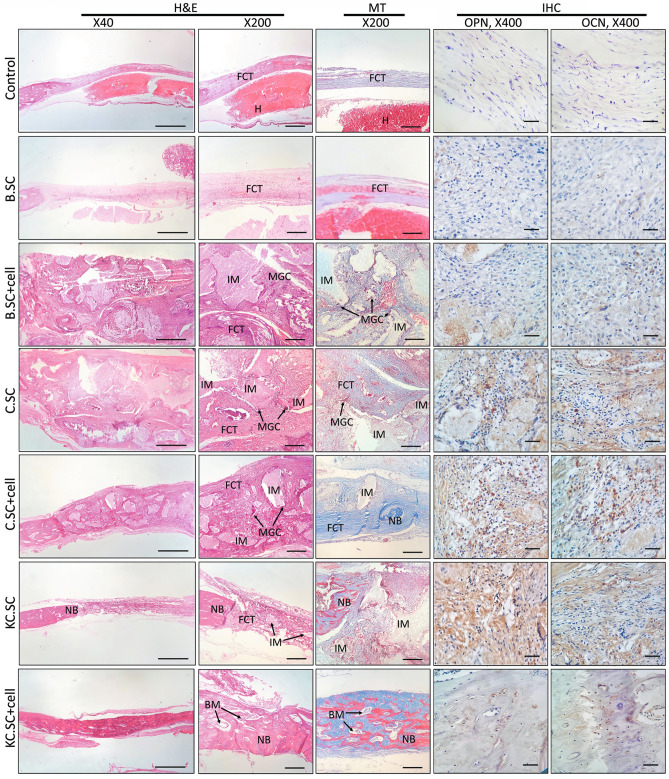
Histopathology images (containing H&E, MT and IHC staining), 12 weeks after implantation.

Figures 7 and 8 include H&E, MT, and IHC results after 4 and 12 weeks post-implantation, respectively. The H&E staining showed that fibrous connective tissue (FCT) filled defect and new bone formation was insignificant in the control group. For defects implanted by B.SC, B.SC + cell, C.SC, and C.SC + cell groups, the implantation sites were substituted with FCT, multinucleated giant cells (MGCs) were visible, and bone reconstructed partly after four weeks post-surgery. In contrast, new bone was formed in KC.SC-implanted groups (with or without BMSC) after 4 and 12 weeks. Despite incomplete osteointegration after four weeks, new bone formation raised after 12 weeks, the defect implanted by KC.SC + cell group was filled entirely by new bone, and bone maturation associated with complete osteointegration occurred after 12-weeks implantation.

Immunohistochemical staining of tissue sections for the OCN and OPN markers confirmed that there were not any differences in expression of OCN and OPN markers between B.SC, B.SC + cell, and control groups. Also, OCN and OPN deposition in C.SC, C.SC + cell, and KC.SC groups were considerably higher than other treatments and control groups. In the KC.SC + cell group, bony islands were observed across the defects.

## Discussion

Since the bone injuries larger than critical size defect cannot be reconstructed, it is important to provide constructs with suitable function for bone regeneration. Optimal physical characteristics, biocompatibility, biodegradation, osteogenesis, and osteointegration are essential features for bone tissue engineering. Also, stem cells, accompanied by biomaterials or tissue-engineered constructs, could improve regeneration of critical-sized bone defects^5,7^. In the current study, we synthesized BMSC-seeded bioglass scaffolds with/without Kaempferol-loaded Zein coating and evaluated them for bone tissue engineering in vitro and in vivo.

Although coating the scaffold with a polymer solution such as Zein would cover the surface and pores, this new structure with the pore size ranging from 200 to 500 µm would be appropriate for cell migration inside the scaffolds^50–52^. Meanwhile, the mechanical strength of coated scaffolds was threefold higher than that of uncoated scaffolds which was parallel to other published reports^27,28,53,54^. Bioactivity analysis showed that HCA crystals covered the surfaces of all scaffolds confirming the stimulating effect of Zein coating on bioactivity due to the presence of proper chemical groups as nucleation sites for HCA deposition^27,28,55^. Besides, attachment and proliferation of BMSC cells on Zein-coated and bare scaffolds proved that Zein hydrophobicity did not have a negative effect on cell functionality^34,56^.

The BMSCs are good cell sources for bone regeneration because of their self-renewal and multipotential differentiation capacity^57^. They are positive for representative expression markers such as CD29, CD90, and CD44, whereas they are negative for hematopoietic markers including CD45, CD11, and CD34^58^.

Alkaline phosphatase (ALP) is a metalloenzyme that encodes various genes in different tissues. ALP has serine phosphate in its active site and can react with O–H in alkaline pH leading to the release of inorganic phosphate^59^. ALP activity is known as an osteogenesis indicator, and early marker of the osteoblast differentiation^35^. The MSCs exude ALP in the early stage of osteo-differentiation and has a positive effect on the beginning of calcification and formation of calcium phosphate clusters by interacting free phosphate with Ca^2+^^60,61^. Our results confirmed the release of ALP increased in all experimental groups, especially KC.SC after 7- and 14-days treatment confirming osteo-differentiation onset in all groups. Further, ALP is a differentiation characteristic whose higher activity points to the transition of BMSCs towards osteoblast maturation^60^. According to Fig. 3b, Zein coating (C.SC sample) caused 35.3 and 6.2% higher activity in ALP compared with the B.SC after 7 and 14 days, respectively. Kaempferol loading (KC.SC sample) had similar but significant trends for ALP activity compared with B.SC (323.5 and 140.6%) and C.SC (213.0 and 126.6%). At Day14, B.SC and C.SC scaffolds could significantly enhance ALP activity compared to Day7 (88.3 and 47.8%, respectively), but ALP activity of KC.SC (7.0%) was not significant. Osteogenic differentiation of stem cells includes three phases: proliferation, ECM maturation, and mineralization. Collagen type 1 gene (COL1) is an early-stage marker that is expressed after proliferation and at the beginning of ECM maturation^60^. The qPCR data revealed that C.SC and KC.SC groups could highly upregulate COL1 after 21 days (225.2 and 44.8%, respectively, compared with B.SC sample) confirming BMSCs transition from proliferation phase to maturation phase^62^. Other osteogenic genes including osteocalcin (OCN) are induced at the beginning of the mineralization stage. Osteocalcin is a late marker of differentiation that acts as a modulator for apatite growth and regulates apatite calcium-binding protein^60^. Considering expression of OCN in highly differentiated cells^60,63^, the greater expression of OCN on Day21 (162.0 and 478.7% for C.SC and KC.SC, respectively, compared with B.SC sample) compared with Day14 (90.0 and 180.0% for C.SC and KC.SC, respectively, compared with B.SC sample) and ALP activity suggested that BMSCs started mineralization and calcification phase^63,64^.

Zein as a biocompatible and biodegradable polymer has osteogenic capability^5^, and can improve bone formation^35,38^. By having no negative effect on the surface adhesion and proliferation of mesenchymal stem cells (MSCs), its osteoconductive properties stimulates MSCs to differentiate towards osteoblasts in the presence of dexamethasone^34^. Zein can enhance the expressions of osteogenic genes such as ALP, COL1 and OCN^36^. In vitro studies about Kaempferol action on osteogenesis have proved that it causes upregulations of various specific genes such as ALP, COL1, OCN, OSX (osterix), BMP-2 (bone morphogenetic protei-2), OPN (osteopoetin), induces mineralization of osteoblasts and calcium deposition, and provokes formation of bone nodules. It stimulates the differentiation murine preosteoblastic MC3T3-E1 cells, and improves their ALP activity and calcification. Bone formation occures through the intramembranous or endochondral ossifications during which MSCs directly or indirectly differentiate into osteoblasts to form finally mineralized bone matrix. Kaempferol can modulate different matrix metalloproteinases to commit MSCs to the osteoblastic lineage by upregulating ALP, Runx-2, OSX and OCN, and downregulating PPAR-γ to prevent adipogenesis^41^. According to Adhikary et al., the osteogenic action of Kaempferol is owed to the BMP-2 upregulation resulting in osteoblast proliferation, and exhibiting high expressions of ALP, OSX, OCN, Runx-2, and COL1 in dexamethasone-induced rat calvarial osteoblasts^65^.

Three-dimensional µCT presented more osteogenic activity for cell-loaded scaffolds compared with cell-free scaffolds. Although all scaffolds with or without BMSC were osteoconductive and osteoinductive, new bone regeneration was significant in the center and sides of defects filled with KC.SC + cell after 12 weeks agreeing with in vitro experiments. In bone repair, biomaterials act as a platform to support stem cell adhesion and proliferation. BMSCs when exposed to chemical compounds, physical stimulations, and surface of biomaterials differentiate into osteogenic cells by releasing matrix components promoting calcification, and forming new bone. Meanwhile, growth factors such as BMP-2 are secreted from committed cells and have a positive effect on attracting, proliferating, and differentiating of mesenchymal progenitor cells resulting in formation of bone tissue in vivo^66^. Our results were parallel with the study accomplished by Mazaki et al. in which bone mineral density of lumbar spine increased by Kaempferol treatment^67^. Moreover, As explained previously^61,68^, periosteal osteoprogenitor cells invade the defect site and cause new bone formation at the peripheral area and on the surface of scaffolds.

As reported earlier^69^, there is a direct correlation between the number of osteoblasts and new bone formation. The histomorphometric analysis showed that osteoblasts, osteons, and osteocytes in KC.SC + cell group were more than other groups after 12 weeks post-surgery. And, new bone formation was the highest and the fibrous connective tissue was the lowest in KC.SC + cell group after 12 weeks.

Monocyte progenitors could differentiate into multinucleated giant cells (MGCs) whose occurrence is enhanced after bone failure, chronic inflammation, tumor formation, and granulomatous disease. Macrophage is an important type of MGCs that is produced in response to infection. Histopathology results showed that MGCs could adhere to scaffold surface and contribute to inflammation and regeneration^70^. Scaffold degradation depends on macrophages' activity that results in polymer degradation and ion release providing a suitable environment for new bone formation. Further, infiltration of macrophages into the implantation site can cause an inflammatory reaction^70,71^. In the current study, defect sites implanted by all experimental groups with or without BMSCs underwent FCT and MGCs accumulation after four weeks post-implantation that could be a symptom of implant-related inflammation. However, macrophages were not seen in KC.SC + cell group after 12 weeks indicating termination of inflammatory process at this time. It could be concluded that implant degraded and osteointegration and remodeling progressed after 12 weeks post-implantation^72^. In the control group, FCT filled the defects and new bone formation was negligible. New bone formation increased in KC.SC but fibrous tissue between the implant and host bone showed that osteointegration was incomplete at both time points^73^. Based on our findings, BMSCs and Kaempferol as an osteogenic agent in KC.SC + cell group could improve new bone formation and osteointegration compared with other groups.

Apart from differentiation capacity into different cell lineages, research findings present that mesenchymal stem cells display broad immunomodulatory properties and can suppress immune rejection^5,7^. So, better bone formation in BMSC-loaded scaffolds might be caused by immunological and biological roles of BMSCs. Aligned with Tu et al.^5^, administration of BMSCs not only supply osteogenic cell source for bone regeneration but also provide growth factors having a positive effect on differentiation and new bone formation.

The immunohistochemical assessment did not show any differences in expression of OCN and OPN markers between B.SC, B.SC + cell, and control groups. However, the number of OPN- and OCN-positive cells in C.SC, C.SC + cell, and KC.SC was considerably higher than expressions of those genes in the control group. It is believed that OCN produced by osteoblasts is needed for mineralization and calcium ion homeostasis^74^. OCN stimulates the maturation and migration of osteoclast precursors, and contributes to the formation and maturation of hydroxyapatite crystals. It is not expressed in osteoblasts at the very early stage of the mineralization process, and detected in the bone matrix after significantly progressed mineralization. Due to very low expression in the early stages of osteoblast differentiation, OCN is considered as a marker of mature osteoblasts^75^. In the C.SC, C.SC + cell, and KC.SC groups, the OCN-positive cells spread throughout all the regions homogeneously showing formation of mineralized ECM. Mature bone tissue and osteon formation in KC.SC + cell group was aligned with histomorphometric analysis; therefore, osteogenesis process finished, OCN decreased, and bone islands formed.

## Conclusion

In this study, the osteogenic potential of porous 58S-based scaffolds including bare, Zein-coated, and Kaempferol-loaded scaffolds was investigated in vitro and in vivo. According to in vitro and in vivo findings, 58S-based scaffolds coated with Kaempferol-loaded Zein with BMSCs could promote osteogenesis resulting in better ALP activity, bone-specific gene expression, and bony island formation. So, combining physical properties of coated scaffolds with the osteogenic effect of Kaempferol and BMSCs may represent a new strategy for bone regeneration and provide a more effective approach to repairing critical-sized bone defects.

## Materials and methods

All methods were carried out in accordance with relevant guidelines and regulations.

### Scaffold preparation and engineering characterization

The protocols for the synthesis of 58S bioactive glass, scaffold preparation and characterization were reported in our previous article^44^. Briefly, the 58S bioglass was synthesized by mixing tetraethyl orthosilicate (TEOS, 39.2% (v/v)), triethyl phosphate (TEP, 1.7% (v/v)), and calcium nitrate tetrahydrate (Ca(NO_3_)_2_⋅4H_2_O, 26.4% (w/v)) in H_2_O. After keeping three days at 25 °C for gelation, the mixture was aged at 60 °C, and dried at 120 °C for 24 h. The prepared 58S bioactive glass was characterized by differential thermal analysis (DTA), thermogravimetric analysis (TGA), X-ray diffraction (XRD), and Fourier-transformed infrared spectroscopy (FTIR). Then bioactive scaffold was made by immersing polyurethane foam into aqueous slurry of 58S (40% (w/v))-poly vinyl alcohol (PVA, 5% (w/v)), followed by heating at 350 °C for 30 min to burn the foam and 1250 °C for three hours to sinter the scaffold body. In the following, prepared scaffold was coated with Zein solution to increase mechanical strength, and loaded with Kaempferol to improve its osteogenic activity. Full structural, surface morphology and mechanical evaluation, assessment of bioactivity and degradation, drug release, and surface cell attachment were carried out. In current study, finally approved 58S scaffold coated with Zein-Kaempferol (7% (w/v)-400 µM) was used for in vitro and in vivo studies.

### BMSCs extraction and characterization

To isolate BMSC cells, rats were sacrificed based on the protocol approved by Ethics Committee of Tehran University of medical sciences (IR.TUMS.VCR.REC.1396.4511), and their bone marrow was aspirated by flushing Dulbecco’s Modified Eagle’s Medium (DMEM, high glucose, Thermo Fisher Scientific, USA). After centrifuging for 10 min at 2000 rpm, supernatant was discarded, and cell pellet was washed by phosphate-buffered saline (PBS) for five minutes at 2000 rpm. Then, the cell pellet was suspended in DMEM supplemented with 20% FBS (Fetal bovine serum, Thermo Fisher Scientific, USA) and 2% penicillin/streptomycin (Thermo Fisher Scientific, USA), and transferred to a 6-well plate (about 4 × 10^4^ cell/well). After incubation in common culture conditions for 24 h, supernatant containing non-adherent cells was replaced with fresh 20%FBS-DMEM, and culture medium was changed every two days. Upon reaching 70–80% confluence, BMSCs were trypsinized, transferred to a 25 cm^2^ culture flask, and subcultured up to the third passage^76,77^.

To characterize BMSCs by flow cytometry, the cells (at passage three) were trypsinized and incubated with FITC-conjugated antibodies such as CD90-FITC, CD44-FITC, CD34-FITC, and CD45-FITC (Abcam, UK). Stained cells were washed with PBS, resuspended in Magnetic-activated cell sorting (MACS) buffer, and characterized by flow cytometer (FACCS Calibure, BD bioscience San Jose, CA, USA).

### In vitro study

To evaluate the osteogenic capacity, biological features of the scaffolds including bare scaffolds (B.SC), Zein-coated scaffolds (C.SC), and Kaempferol-loaded C.SC (KC.SC) were assessed by analyzing specific enzymatic activity, staining deposited minerals by cells, and measuring gene expression.

#### Calcium deposition assessment

Alizarin red staining was accomplished to detect calcium deposition in the extracellular matrix after 21 days. Because of the presence of calcium in scaffolds, it was difficult to distinguish calcium deposition in ECM. Thus, BMSCs were cultured in the plate, and conditioned media (10% FBS-supplemented DMEM containing 0.1 mg/mL of each scaffold) were added. After 21 days, media was removed; the BMSCs were washed with PBS, and fixed with 4% paraformaldehyde (PFA) for 40 min at room temperature. Then, PFA was pulled out and replaced with alizarin red solution (2% (w/v) in deionized water, pH 4.1–4.3). After incubation at 4 °C for 40 min, the stained BMSCs were observed under an inverted microscope (Labomed, USA).

#### Alkaline phosphatase (ALP) activity

Colorimetric ALP assay kit (Abcam, USA) was applied to measure the alkaline phosphatase activity according to the manufacturer’s instruction. Briefly, supernatants of the BMSCs cultured on the different scaffolds were accumulated every two days for 7 and 14 days. Then, 80 µL of supernatant was incubated with 50 µL of p-nitrophenyl phosphate (pNPP, Sigma-Aldrich, USA) at 37 °C for one hour in darkness, and absorbance was measured by a microplate reader (BioTek, USA) at 405 nm.

#### RNA isolation and gene expression evaluation

After treating BMSCs with conditioned media of different scaffolds for 14 and 21 days, BMSCs were washed by PBS, trypsinized, and total RNA was extracted using RNA extraction kit (Yekta Tajhiz, Iran). RNA integrities and purities were estimated using a Nanodrop spectrophotometer (Thermo scientific, USA) by considering the ratios of 260/280 and 260/230 about 2 as purity and integrity criteria. Complementary DNA (cDNA) was synthesized using RT-PCR Pre-Mix BioFact kit (BIOFACT, South Korea), and amplified by Corbett Rotor-Gene 6000 (Qiagen, UK) using HOT FIREPol EvaGreen qPCR Mix Plus (Solis BioDyne, Stonia). The concentration of cDNA was considered the same for all samples to compare the results correctly. Table 1 contains sequences of forward and reverse primers including collagen1 (COL1) and osteocalcin (OCN) as target genes and glyceraldehyde 3-phosphate dehydrogenase (GAPDH) as control gene. The expression of COL1 and OCN genes for treated BMSCs were analyzed and compared with control sample (untreated BMSC) through 2^-ΔΔCt^ method.

**Table 1:** Primer sequences of target and control genes used for real time PCR reactions.

Target gene	Sequence		Primer length	Product length	Tm
GAPDH	Forward	GATCAAGATCATTGCTCCTCCTG	23	170	58.8
	Reverse	CAGCTCAGTAACAGTCCGCCTAG	23	170	59.2
COL1A1	Forward	GGACACTACCCTCAAGAGCCTG	22	129	61.9
	Reverse	TACTCTCCGCTCTTCCAGTCAGA	23	129	61
Osteocalcin	Forward	ACAAAGCCTTCATGTCCAAGCA	22	217	60.9
	Reverse	GACATGCCCTAAACGGTGGT	20	217	61.2

### In vivo assessments

#### Implantation procedures

All animal experiments were approved by Committee on Animal Care at Faculty of Veterinary Medicine at the University of Tehran (IR.TUMS.VCR.REC.1396.4511). Also, the ARRIVE guidelines were considered to perform all experiments in this study. Seventy male Sprague Dawley rats with an average weight of 250–300 g were used in this experiment. Scaffolds were divided into six groups: bare scaffold (B.SC), cell-seeded B.SC (B.SC + cell), Zein-coated scaffold (C.SC), cell-seeded C.SC (C.SC + cell), Kaempferol-loaded C.SC (KC.SC), and cell-seeded KC.SC (KC.SC + cell). Two days before surgery, the total BMSC numbers of 5 × 10^5^ was seeded on each sample. The rats were anesthetized, shaved, and sterilized for surgery, and a circular defect (diameter of 8.0 mm) was created in calvarium under steady DPBS (Dulbecco’s phosphate-buffered saline) pouring to prevent overheating. Then the scaffolds were implanted in the calvarium defects. To prevent infection, enrofloxacin 5% was injected into rats before surgery, and added to drink water (0.5% (v/v)) three days after surgery. Considering calvarium defect without any scaffold as control group, ten rats were observed in each experimental group for 4 and 12 weeks (five rats for each time point).

#### Radiological and three-dimensional micro-computed tomography (3D µCT) evaluation

After 4 and 12 weeks post-implantations, animals in each group (five rats each time point) were sacrificed by overdosed inhalation of carbon dioxide. Tissue samples were collected by preserving surrounded calvarium bone. Primary evaluation of bone regeneration was conducted by digital radiography (Kodak direct view CR850, Japan) immediately. The 12-weeks related samples were fixed by formalin (10%, pH 7.26) and 3D µCT was performed to analyze 3D bone regeneration using a desktop 3D µCT instrument (LOTUS-NDT, Behin Negareh.Co, Iran). Samples were sealed at a micro-focus X-ray tube, and scanning was performed by setting suitable parameters (voltage of 80 kV, current of 80 µA, best time of 2 h) resulting in resolution of about 10 µm.

#### Tissue processing and staining

The collected and fixed tissue samples were decalcified with 14% ethylenediamine tetra acetic acid (EDTA) solution for four weeks. Then, they were dehydrated in different grades of ethanol solutions (70, 90, and 100%), cleared with xylene, embedded in paraffin, and cut into sections with thickness of 5 μm using microtome. All sections were fixed on histological slides, and stained with hematoxylin–eosin (H&E) and Masson's trichrome (MT). The stained samples were evaluated and imaged using an Olympus BX51 light microscope (Olympus, Japan).

#### Histomorphometric analysis

Histomorphometric analyses of random H&E images were conducted by computer software Image-Pro Plus® V.6 (Media Cybernetics, USA) to quantify the percentage of new bone (NB) and fibrous connective tissue (FCT) in the defect area using Eq. (1):

1 $${\text{NB}}\;{\text{or}}\;{\text{FCT}}\% = \frac{{{\text{NB}}\;{\text{or}}\;{\text{FCT}}\;{\text{area}}}}{{{\text{original}}\;{\text{total}}\;{\text{defect area}}}} \times 100$$

Whole defect were observed by microscope to check new bone formation and the FCT presence. To measure the numbers of cells (fibroblasts, osteoblasts, fibrocytes, osteoclasts, osteocytes), and osteons, four microscopic spans (at 400× magnification) were randomly selected at the margin and center of the defect^78^. For avoiding bias, the samples’ areas were chose and microscopic images were taken by a person who did not know anything about the study.

#### Immunohistochemical analysis

The histological slides of different groups were analyzed for expression of osteocalcin and osteopontin antibodies. The slides were incubated in citrate buffer solution at 60 °C overnight, and blocked with 1% hydrogen peroxide/methanol at room temperature for 30 min. Then, the slides were incubated with primary anti-osteocalcin and anti-osteopontin antibodies (Abcam, USA) at 4 °C for 12 h. The color reaction was developed with ready to use 3,3′-diaminobenzidine (liquid DAB color solution, Dako, Denmark), and the slides were counterstained with 1% (w/v) hematoxylin (Sigma-Aldrich, USA).

### Statistical analysis

Data was presented as the mean ± standard deviation. After analysis of normality and homogeneity of variance using Kolmogorov–Smirnov test and Levene's test, statistical analysis was done through one-way ANOVA and Student’s t-test at significance of 0.05 (p < 0.05).
